# Retraction: Lumen-Apposing Metal Stent for Pyloric Stricture in Crohn’s Disease: A Case Report

**DOI:** 10.7759/cureus.r179

**Published:** 2025-04-11

**Authors:** Ayah Obeid, Gursimran S Kochhar

**Affiliations:** 1 Internal Medicine, St. Luke's University Health Network, Bethlehem, USA; 2 Gastroenterology, Hepatology, and Nutrition, Allegheny Health Network, Pittsburgh, USA

This article has been retracted by the Editors-in-Chief at the request of the authors due to their failure to obtain patient consent prior to publication. Due to the resulting patient privacy concerns, the article content has been removed.

